# Supplementary material for: Patterns of Intron Gain and Loss in Fungi
Source: PLoS Biol. 2004 Nov 30;2(12):e422. doi: 10.1371/journal.pbio.0020422 (PMC532390; doi:10.1371/journal.pbio.0020422)
Supplement: Table S1 — Also available at http://genes.mit.edu/NielsenEtAl/. (4.3 MB ZIP). [file pbio.0020422.st001.zip › NielsenEtAl/html/701.html]

AN4042.1.NCU05278.1.MG06047.1.FG01959.1


```
 CLUSTAL W (1.82) Multiple Sequence Alignments - Introns Inserted


Sequence 1: MG06047.1	541 aa
Sequence 2: FG01959.1	536 aa
Sequence 3: NCU05278.1	533 aa
Sequence 4: AN4042.1	534 aa
Alignment Length: 547 aa
Number Identitical Residues: 266 aa
Alignment Score (without introns) 13920


MG06047.1 	MASVAVGVANATAAFTSPLASYVPSNVALPPQLEYVLGNVIETVSNAGFWTILGTVVLML
NCU05278.1	-------MASNATSFTSPLASAKLGTVAVPPQLEY----VIDTISQASGWTIVFTLLAVL
FG01959.1 	----MEAVNSTTSGFSSVLAGTKYANVNIPPQIDY----VIEAVSNAGVWTWVFTLVALC
AN4042.1  	-------MAEINGSFVSPAA-----DATVFPQVFQPAGLIADFLNGLTLWKTLATLFALA
          	       : .   .* *  *      . : **:    . : : :.    *. : *:. : 

MG06047.1 	AIYDQF1SYQRSKGNIVGPRFKEPFIGPFLQSMNPKWEEYYGKWTSGPLSCVSVFHK~YA
NCU05278.1	VAWDQI1KYVLNKGTIAGPSWKIPFIGPFLESLDPKFEEYYAKWLSGPLSCVSVFHK2FV
FG01959.1 	IAYDQI1AYIVRKGPIVGPAMKIPFIGPFLDSMDPRFDGYHAKWSSGPLSCVSIFHK2FV
AN4042.1  	VVYDQF1RYIYLKGAIVGPAWKLPFMGPFLQSVNPKFHEYKAKWDSGELSCVSVFHK2FV
          	  :**:  *   ** *.**  * **:****:*::*::. * .** ** *****:*** :.

MG06047.1 	YTFPHSKTVTELLN-----M0PCVVDVAPKLLGHDNWVFLDGKAHVDFRKGLNGLFTRKA
NCU05278.1	VIASTRDMARKVFNSPSYVK~PCVVDVAHKLLGADNWVFLDGPAHVEFRKGLNGLFTRRA
FG01959.1 	VIASTRDMARKVFNSPAYVK~PTVVDVAPKLLGHDNWVFLDGKAHVDFRKGLNGLFTRKA
AN4042.1  	VIASTRDMSRKIFNSPTYVK~PCVVDAAHKLLGKTNWVFLDGKEHVDFRKGLNNLFTRQA
          	   .  .   :::*:.:    * ***.* ****  *******  **:******.****:*

MG06047.1 	LECYLPGQEDVYNRYFKKMVQITKDAGGKPVPFMVEFREIITAVSCRTFVGHYMSDETVR
NCU05278.1	LEIYLPGQEEVYNRYFKSFLEITKNAGGKPVPWMPHFREVITAVSCRSFVGHYISDEAVK
FG01959.1 	LELYLPGQEEAYNTYFKHFLKMTKDAGGKPVPFMHEFREVMCAVSCRTFVGHYISDEAVT
AN4042.1  	LSCYLPRMEEVYNDYYARFLKKSKNNNYKPTPWMPEFRDLMCAVSCRTFVGHYISDEAID
          	*. ***  *:.** *:  ::: :*: . **.*:* .**::: *****:*****:***:: 

MG06047.1 	RIAVDYFYITEALELVNFPIILPFTKTWYGKKAADMVLAEFSKCAAKSKARMNADGEVTC
NCU05278.1	KIADDYYLITAALELVNFPIIIPYTKTWYGKKAADMVLAEFAKCAAKSKVRMAAGGEATC
FG01959.1 	KIAEDYYLITAALELVNLPVILPYTKSWYGKKAADMVLAEFSKCAAKSKVRMAAGGEVTC
AN4042.1  	KISVDYYNITAALELVNFPIILPFTKTWYGKKAADMVLDEFAKCAAKSRARMAAGGEISC
          	:*: **: ** ******:*:*:*:**:*********** **:******:.** *.** :*

MG06047.1 	IMDAWIQQMVLSQRWRDAEAAGTITDDMEKPNPLLRDFTDYEIAQTLFTFLFASQDATSS
NCU05278.1	IMDAWIKQMIDSKKWREAVASG-NTEGLEKPTHLLRDFTDYEISQTIFTFLFASQDATSS
FG01959.1 	IMDAWILSMIQSERWRKAEEKG-EPHNVEKPSPLLRMFNDYEISQTIFTFLFASQDATSS
AN4042.1  	IMDAWIKAQLDSAKYREKIAKGIEVDSSEKPPQVLRDFTDYEVSQTIFTFLFASQDATSS
          	******   : * ::*.    *   .. ***  :** *.***::**:*************

MG06047.1 	AATYMFQIMAQHPEVLDRVREENYNVRNGDINARVSLEQLESMKYTRAVVKELLRYRPPV
NCU05278.1	AATWLFQIMAQRPDVLDRVREENLKVRNGDIHAPINMEQLESMTYTRAVVRELLRYRPPV
FG01959.1 	AATWLFQVTAQRPDVLDRVREENIKIRNGDPNAPLTMDQLESLTYTRAVVRELLRWRPPV
AN4042.1  	ACTWLFQLMADRPEILDKVREENLRLRNGDVNAPLTMDLLDSMTYTRAVVKETLRYRPPV
          	*.*::**: *::*::**:***** .:**** :* :.:: *:*:.******:* **:****

MG06047.1 	IMVPYVTKKAFPITPEYTAPKG1SMLIPTTYMALRDPEVYDRPDEFDPERYYTGDAEEKG
NCU05278.1	LMVPYLVKKAFPITEDYTIPKG~SMVVPTTYMALRDPEVYPNPDYFDPERYYSGDAEAKG
FG01959.1 	IMVPYVTKKAFPLTDDYTVPKG1SMLIPTTFMALHDPEVYDNPSHFDPERYYSGDAEEKG
AN4042.1  	IMVPYIAKKDFPITDKITVAKG~SMIIPSVYPATRDEEAYPNADSFDPDRWITGTAEQHP
          	:****:.** **:* . * .** **::*:.: * :* *.* ... ***:*: :* ** : 

MG06047.1 	QKNFLVFGTGPHYCLGQHYAQLNLALFVGMASLQLDWKHHATPLSEEIQVFATIFPK0DH
NCU05278.1	AKNYLVFGVGPHYCLGQHYAQLNLALFVGKASLLLDWKHHPTPLSEEIKVFATIFPK0DD
FG01959.1 	SKNYLVFGTGPHYCLGQVYAQLNLALMIGKASVMLDWKHHATPKSEEIKVFATIFPM0DD
AN4042.1  	-KNFLIFGTGPHYCLGQTYAVLNLMAMIGKASMEMDWVHTPTPQSEEIKVFATIFPQ0QP
          	 **:*:**.******** ** ***  ::* **: :** * .** ****:*******  : 

MG06047.1 	CPLTFDWRKWN-
NCU05278.1	CPLTFEERKW--
FG01959.1 	CPLTFEERKW--
AN4042.1  	GDLDELCHSMAL
          	  *    :.
```
